# Malaria Vaccine Rollout in Nigeria: Current Status and Challenges

**DOI:** 10.4269/ajtmh.26-0371

**Published:** 2026-08-27

**Authors:** Samuel Adeniyi Oyegbade, Samson Olusola Adeboye, Gbotemi Jerry Oni, Abosede Peace Oyegbade, James Joy Oziohu, Zawu Yoowa Duyann, Oluwatoyin Comfort Owoyemi

**Affiliations:** ^1^ Department of Biological Sciences, College of Science and Technology, Covenant University, Ota, Nigeria;; ^2^ Africa Centre of Excellence for Mycotoxins and Food Safety (ACEMFS), Federal University of Technology, Minna, Nigeria;; ^3^ Ace Medicare Clinics, Ota, Nigeria;; ^4^ Department of Biochemistry, University of Ilorin, Ilorin, Nigeria;; ^5^ University of Liberia Fendall Campus, Monrovia, Liberia;; ^6^ Div Medical Services and Hospital, Adekunle Fajuyi Cantonment, Ibadan, Nigeria

## Abstract

Malaria remains a leading cause of morbidity and mortality in Nigeria, particularly among children under 5 years of age, despite ongoing control efforts. The introduction of RTS,S/AS01, and R21/Matrix-M vaccines provides a promising complementary prevention strategy. This systematic review assessed malaria vaccine rollout in Nigeria, focusing on implementation, awareness, acceptance, willingness to pay, and health system readiness. A comprehensive search of PubMed, ScienceDirect, Google Scholar, African Index Medicus, and gray literature (2015–2026) identified 12 eligible studies. Caregiver acceptance ranged from 76.6% in Lagos to 95.6% in Owerri, and exceeding 90% in Enugu, Ondo State, and Gombe. Among health care professionals, only 54.1% of pharmacists were willing to vaccinate their children. Caregiver awareness ranged from 9.7% in Lagos to 72.1% in Enugu. Physicians demonstrated higher adequate knowledge (68.9%) than nurses (35.9%), with 55.5% of nurses having only heard of the vaccine. Pharmacists expressed concerns about vaccine failure (53.6%), adverse effects (63.4%), and storage conditions (77.8%). Willingness to pay was moderate; median willingness in Enugu was USD $0.82 per dose; 71.2% of Lagos caregivers expressed willingness to pay; and 90% of respondents nationally preferred government-funded vaccination. Health system readiness was critically inadequate, with overall facility preparedness scores ranging from 33% to 51%. Advocacy and social mobilization were at 0% across all three facilities, and monitoring and supervision ranged from 0% to 33%, all far below the WHO-recommended 95% threshold. Strengthening health systems, targeted communication, and sustainable public financing are essential for a successful nationwide malaria vaccine rollout.

## INTRODUCTION

Malaria remains one of the leading causes of preventable morbidity and mortality worldwide. In 2024, an estimated 282 million malaria cases and 610,000 deaths were reported globally, with the vast majority occurring in the WHO African Region.^1^ It is known that the disease is more prevalent among groups of people who are highly susceptible to it, such as pregnant women and children younger than 5 years old. In 2024, children younger than 5 years old made up 75% of malaria mortality in the WHO African Region, and 13 million out of 36 million pregnant women in the region suffered from the disease.^1^ Malaria is transmitted through the bites of infected female *Anopheles* mosquitoes carrying *Plasmodium* parasites. Among the five major *Plasmodium* species that infect humans, *Plasmodium falciparum* is responsible for the most severe forms of the disease, including severe anemia, cerebral malaria, and death.^2^^,^^3^

Nigeria bears the greatest malaria burden globally. In 2024, the country accounted for 24.3% of all malaria cases, 30.3% of malaria-related deaths worldwide, and 38.6% of global malaria deaths among children under 5 years of age.^1^ With more than 97% of the population living in areas of malaria transmission and the highest number of at-risk newborns globally, malaria remains a major public health and socioeconomic challenge in the country.^4^

The WHO recommendation of the RTS,S/AS01 and R21/Matrix-M malaria vaccines represents a major milestone in global malaria prevention. RTS,S/AS01 was recommended by the WHO in 2021 after successful pilot implementation in Ghana, Kenya, and Malawi, whereas R21/Matrix-M received a WHO recommendation in 2023 after demonstrating an efficacy exceeding 75% in Phase IIb clinical trials, surpassing the WHO’s preferred efficacy target.^5^ Nigeria became the second country after Ghana to grant regulatory approval for the R21/Matrix-M vaccine in April 2023 and received its first shipment of the vaccine in October 2024, initiating phased implementation in Bayelsa and Kebbi States.^4^ By the end of 2024, Nigeria had become one of 17 African countries to incorporate malaria vaccination into its routine childhood immunization program.^6^ In addition to proving implementation preparedness, the efficacy of the malaria vaccine is another important aspect that justifies its incorporation into the malaria control strategy of Nigeria. As seen from the results of the trials of R21/Matrix-M in Phase IIb, the level of efficacy was estimated at 77%, whereas in the Phase III trials, it ranged between 66% and 75%.^5^ The R21/Matrix-M vaccine has demonstrated higher efficacy than RTS,S/AS01, which has an efficacy that has been reported to vary with age, transmission setting, and duration of follow-up, ranging from approximately 26% to 56% in clinical trials.^7^ However, these efficacy estimates should be interpreted with caution because the two vaccines were evaluated in separate clinical trials conducted in different epidemiological settings rather than in direct head-to-head comparisons. Consequently, although the available evidence suggests that R21/Matrix-M may achieve higher efficacy, the precise comparative performance of the two vaccines remains uncertain.

Effective deployment of vaccines is hampered by several persistent constraints, such as the lack of cold-chain infrastructure, limited capacity of the health workforce, inequitable immunization coverage, and logistical challenges.^4^ In addition to that, the existing fragmented research on malaria vaccine awareness, acceptance, and health system readiness for malaria vaccines in Nigeria is geographically limited and has not been synthesized to inform a national-level implementation strategy. This review synthesizes existing evidence on malaria vaccine deployment in Nigeria and provides some recommendations based on the identified gaps and challenges.

## REVIEW DESIGN

The research design for the study was a systematic review based on PRISMA 2020 guidelines.^8^ The purpose of the review was to bring together the empirical evidence available on the current situation of rollout of malaria vaccines in Nigeria, with a specific focus on implementation processes, uptake, acceptance, and challenges. The research team adopted a structured approach in the selection and review of relevant literature, presenting transparent evidence synthesis methods in public health research.

### Data sources and search strategy.

The literature search was carried out in several electronic databases to ensure the inclusion of a broad range of peer-reviewed evidence. The databases searched included PubMed/MEDLINE, ScienceDirect, African Index Medicus, and Google Scholar. Studies published between 2015 and 2026 were considered eligible. The start year (2015) was selected to capture evidence generated after the publication of pivotal Phase III RTS,S/AS01 trial findings and the subsequent transition of malaria vaccines from late-stage clinical evaluation to policy development, pilot implementation, and eventual rollout in Africa. The end year (2026) ensured inclusion of the most recent evidence on the introduction and early implementation of the R21/Matrix-M vaccine in Nigeria.

Gray literature was identified systematically from various sources, including policy papers, guidelines, and reports from the WHO, UNICEF, Gavi, the Nigerian Federal Ministry of Health, and the Nigerian Centre for Disease Control (NCDC). Such evidence was included to capture implementation experiences that may not have been reported in peer-reviewed publications.

The search strategy combined controlled vocabulary (MeSH terms) and free-text keywords, including *malaria vaccines*, *RTS,S/AS01*, *R21/Matrix-M*, *Nigeria*, *rollout*, *implementation*, *delivery strategy*, *uptake*, *acceptance*, *coverage*, *barriers*, and *challenges*. Boolean operators (AND/OR) were used to combine search terms appropriately. Reference lists of included studies and forward citation tracking were also examined to identify additional relevant publications Table 1.

**Table 1:** Search Strings

Database	Search Strategy	Filters Applied
Google Scholar	(“malaria vaccine” OR “malaria vaccination”) AND Nigeria AND (implementation OR rollout OR uptake OR acceptance OR awareness OR coverage OR barriers)	Publication years: 2015–2026; English-language publications. Results were sorted by relevance and screened for eligibility
ScienceDirect	(“malaria vaccine” OR “malaria vaccination”) AND Nigeria AND (implementation OR rollout OR uptake OR acceptance OR awareness OR coverage)	Publication years: 2015–2026; English-language publications; Research articles
PubMed	(“malaria vaccine"[MeSH Terms] OR “RTS,S/AS01"[tiab] OR “R21/Matrix-M"[tiab] OR “malaria vaccination"[tiab]) AND (“Nigeria"[MeSH Terms] OR “Nigeria"[tiab]) AND (“rollout"[tiab] OR “implementation"[tiab] OR “deployment"[tiab] OR “uptake"[tiab] OR “acceptance"[tiab] OR “awareness"[tiab] OR “perception"[tiab] OR “readiness"[tiab] OR “preparedness"[tiab] OR “coverage"[tiab] OR “barriers"[tiab] OR “challenges"[tiab])	Publication years: 2015–2026; English-language publications

### Inclusion and exclusion criteria.

Studies that reported any form of empirical data on the deployment of malaria vaccines were eligible for inclusion. This included randomized controlled trials, observational studies, cross-sectional studies, qualitative studies, and mixed-method studies. In addition, programmatic reports and policy papers with empirical information on vaccine deployment were included. Nonempirical studies were not eligible for inclusion; these comprised literature reviews, commentaries, editorials, and opinion pieces. All studies carried out in countries other than Nigeria were excluded, except if they had specific findings or context relevant to Nigeria. Finally, studies that only examined the immunogenicity and efficacy of vaccines in the laboratory and did not address their deployment or acceptance were not included.

### Data extraction.

**Figure 1.**
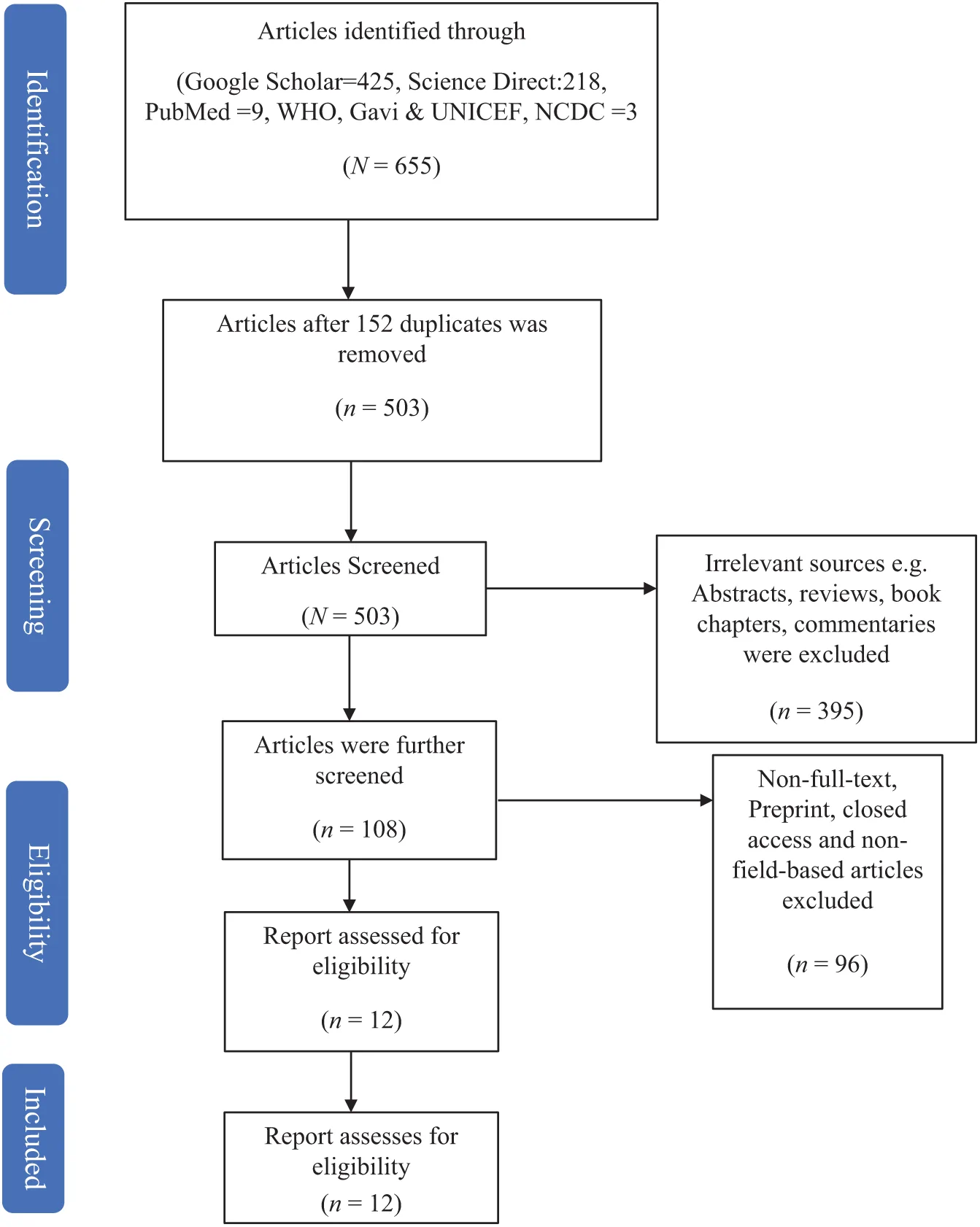
PRISMA flow chart.

Studies retrieved from all databases were imported into Zotero reference management software, where duplicates were removed and records were organized for screening. Title and abstract screenings were subsequently performed within Zotero, followed by full-text assessment of eligible studies. Data extraction was conducted independently by two reviewers using a standardized Microsoft Excel^®^ spreadsheet covering the following parameters: study characteristics (author[s], year, design, and setting); vaccine type (RTS,S/AS01 or R21/Matrix-M); implementation strategy; outcome measures, including vaccine uptake, acceptance, and perception; barriers and facilitators to rollout; and health system factors such as cold-chain capability, workforce availability, logistics, and policy and financing environments. Discrepancies between reviewers were resolved through discussion and consensus. The study selection process is illustrated in Figure 1, and characteristics of included studies are presented in Table 2.

**Table 2:** Characteristics of included studies (*N* = 12)

References	Study Design	Location	Population	Vaccine Focus	Sample Size (*n*)
10	Cross-sectional	Owerri West, SE Nigeria	Caregivers (mothers)	Malaria vaccine (prospective)	500
11	Cross-sectional	Enugu State (UNTH and ESUTH), SE Nigeria	Hospital pharmacists	Malaria vaccine	187
12	Cross-sectional	Enugu Metropolis, SE Nigeria	Mothers	Malaria vaccine	491
2	National cross-sectional	Nigeria (nationwide)	Parents of under-5 children	Malaria vaccine	1,413
13	Cross-sectional	Enugu State, SE Nigeria	Pregnant women and nursing mothers	R21/Matrix-M	310
14	Mixed methods	Ondo State (Ondo West LGA), SW Nigeria	Mothers	Malaria vaccine	359
15	Mixed methods cross-sectional	Gombe LGA, NE Nigeria	Caregivers (PHC users)	R21/Matrix-M	293
16	Cross-sectional	Six geopolitical zones, Nigeria	Nurses	RTS S/AS01 and R21 matrix M	422
17	National online survey	Nigeria (six geopolitical zones)	Physicians	RTS S/AS01 and R21 matrix M	508
18	Facility-based cross-sectional	Northern Nigeria (ABUTH, UMTH, JUTH)	Health facilities/hospital staff	R21/Matrix-M	3 hospitals
19	Cross-sectional	Lagos State, SW Nigeria	Caregivers of under-5 children	R21/Matrix-M	372
6	Policy/program report	Nigeria (Abuja)	National immunization program	R21/Matrix-M rollout	NA

ABUTH = Ahmadu Bello University Teaching Hospital; UNTH = University of Nigeria Teaching Hospital; ESUTH = Enugu State University Teaching Hospital; JUTH = Jos University Teaching Hospital; LGA = Local Government Area; NA = Not Applicable; NE = northeast; PHC = Primary Health Care; SE = southeast; SW = southwest; UMTH = University of Maiduguri Teaching Hospital.

### Methodological quality assessment.

To evaluate the methodological quality of the 11 main studies, the Newcastle-Ottawa Scale (NOS) adapted for cross-sectional studies was used.^9^ The instrument consists of three domains (maximum 5 stars, maximum 2 stars, and maximum 3 stars) for a total of 0 to 10 stars. Those studies scoring 9 to 10 were rated very good, 7 to 8 good, 5 to 6 satisfactory, and below 5 unsatisfactory. All studies were independently reviewed by two reviewers, and disagreements were addressed by discussion. The WHO program report^6^ was excluded from formal quality appraisal because it constitutes a policy document rather than a primary research study.

### Malaria vaccine implementation in Nigeria.

Nigeria received 1 million doses of the R21/Matrix-M vaccine on October 17, 2024, 846,200 procured through Gavi and 153,800 funded by the Nigerian government, commencing rollout on December 5, 2024 in Bayelsa and Kebbi States, which were selected for their high malaria burden.^6^ Kebbi State alone received 595,980 doses targeting 179,542 children age 5 to 15 months, where malaria prevalence in children ages 6 to 59 months stands at 49%, more than double the national figure of 22%.^6^ The vaccine follows a four-dose schedule integrated into Nigeria’s Routine Immunization program at 5, 6, 7, and 15 months, with flexibility for delayed cases.^6^

Implementation is phased, prioritizing children under 5 years of age in high-burden states, with expansion contingent on pilot performance.^4^ Equity remains a central concern, because socioeconomic and regional disparities risk producing unequal vaccine access across Nigeria’s 36 states.^18^ Early field reports indicate high caregiver enthusiasm and no significant resistance in both pilot states.^6^ However, no Nigeria-specific post-introduction effectiveness data are yet available; efficacy estimates are currently extrapolated from Phase III trials demonstrating 75% efficacy under seasonal administration and 68% under year-round dosing in African children.^5^ Real-world effectiveness studies are urgently needed to guide national scale-up.

### Knowledge, awareness, and perceptions of malaria vaccines among caregivers and health care workers.

In the studies included, awareness was measured in most studies using a self-reported measure of whether the participant knew of a malaria vaccine approved for use in Nigeria, based on structured questionnaires. Although some studies focused on general awareness of any malaria vaccine, others specifically focused on the name of the vaccine (R21/Matrix-M or RTS,S/AS01), the estimates below should be interpreted with the understanding of this heterogeneity.

**Figure 2.**
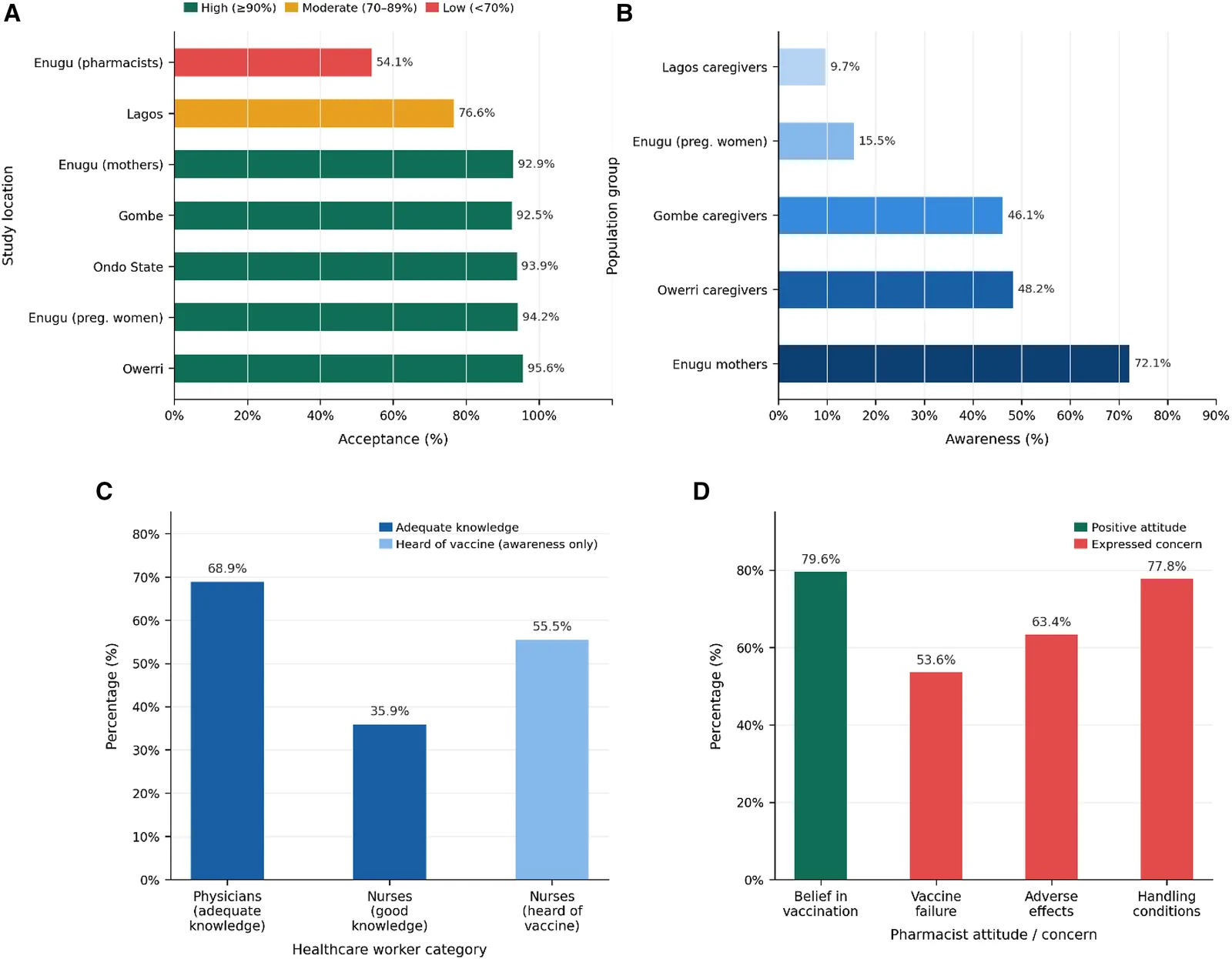
Knowledge, awareness, and vaccine acceptance among caregivers and health care workers in Nigeria. (**A**) Caregiver vaccine acceptance by study location; color denotes acceptance tier (high ≥90%, moderate 70% to 89%, low <70%). (**B**) Malaria vaccine awareness across caregiver population groups, ranging from 9.7% in Lagos to 72.1% in Enugu. (**C**) Health care worker knowledge and awareness by cadre; dark blue = adequate knowledge, light blue = heard of vaccine (awareness only). (**D**) Pharmacist-specific attitudes and concerns regarding the malaria vaccine (Isah et al.^11^); green = positive attitude, red = expressed concern. preg. women = pregnant women.

Malaria vaccine awareness and knowledge were highly variable among people and geographic regions in Nigeria. However, the awareness of the caregivers was generally low, varying from 9.7% in Lagos^19^ to 72.1% in Enugu,^12^ showing wide regional variations. Likewise, moderate awareness was reported in northern Nigeria, with only 46.1% of the caregivers in Gombe State and 48.2% of caregivers in Owerri^10^ being aware of the introduction of malaria vaccines,^15^ and only 15.5% of mothers and pregnant women in Enugu were aware of the malaria vaccine.^13^ These results indicated that there is a disparity in information spread about vaccines across the country (Figure 2).

Awareness and knowledge levels among health care workers are relatively good, but the levels were still suboptimal. The level of knowledge among physicians (68.9%) was considered adequate based on national assessments,^17^ but the nursing population showed significant gaps in knowledge and perception, with a small proportion (35.9%) reporting good knowledge about malaria vaccines, whereas 55.5% had only heard about the malaria vaccine.^16^ Similarly, in southeastern Nigeria, 79.6% of pharmacists expressed belief in vaccination; however, 53.6% had concerns about vaccine failure to protect against malaria, 63.4% had concerns about adverse effects following immunization, and 77.8% had concerns about vaccine handling conditions in Nigeria.^11^

The perception of malaria vaccines was more positive than the level of awareness among both caregivers and health care workers. Positive attitudes to vaccines were reported in several studies, ranging from moderate (55.7%) to high acceptance (98.1%)^19^ in various settings. An interesting paradox was that low awareness did not imply negative perception. Rather, trust in health care systems, perceived severity of malaria, and overall pro-vaccine attitudes seemed to underlie positive perceptions, even in the absence of specific knowledge. Other evidence suggested that knowledge of malaria vaccines and trust in vaccine safety are important factors in predicting vaccine acceptance. In summary, the literature made it clear that although awareness of malaria vaccines varied in Nigeria, especially among parents, there was a positive attitude toward their use. It will be important to focus on enhancing health communication and education to improve uptake of the vaccines.

### Vaccine acceptance, hesitancy, and willingness to pay.

There was generally a high level of willingness to vaccinate children against malaria in all studies carried out in Nigeria, suggesting there was good potential for the uptake of malaria vaccines if they were effectively deployed. The acceptance of caregivers was reported to vary between 76.6% in Lagos^19^ to 95.6% in Owerri,^10^ with the majority of studies reporting over 90%. This high acceptance was also seen in other geographical areas of Nigeria, such as Enugu (92.9%,^12^ Gombe [92.5%],^15^ and Ondo State [93.9%]^14^), indicating a positive attitude toward malaria vaccination in different geographical settings. Comparatively lower acceptance rates were reported among health care professionals, with 54.1% of pharmacists willing to vaccinate their children.^11^

Levels of malaria vaccine acceptance reported across studies were presented in Table 3. In all studies, good knowledge, perception, and trust in vaccine safety and effectiveness were strong correlates of acceptance. Willingness to vaccinate was significantly boosted by a high level of knowledge and a positive perception of the vaccine, as demonstrated in Ondo State, where both knowledge (adjusted odds ratio [AOR] = 2.5) and perception (AOR = 8.7) were significant factors that influenced willingness to vaccinate.^14^ Likewise, vaccine safety and efficacy were important factors that influenced willingness, as a large proportion of respondents in Gombe State (73.0%) indicated safety and efficacy as their motivating factors for acceptance.^15^ Socioeconomic status is also a factor that affected acceptance. In certain contexts, lower socioeconomic groups were less inclined to accept vaccines.^12^

**Table 3:** Level of vaccine acceptance in Nigeria

Location	*N*	Acceptance (%)	Key Predictors/Findings	References
Owerri SE	500	95.6	Perception strongly predicted uptake (χ^2^ = 144.52, *P* <0.0001)	10
Enugu SE	491	92.9	Low SES is associated with lower acceptance (AOR = 0.2, 95% CI: 0.1–0.5)	12
Enugu SE	187	54.1	Education and age significantly influenced acceptance (*P* = 0.017, *P* = 0.014)	11
Enugu SE	310	94.2	High acceptance; age and health insurance influenced willingness to pay; insured respondents had lower willingness to pay (OR = 0.417, 95% CI: 0.19–0.92)	13
Ondo SW	359	93.9	Knowledge (AOR = 2.5) and perception (AOR = 8.7) increased willingness	14
Gombe NE	293	92.5	Trust in safety/efficacy (73%) major driver	15
Lagos SW	372	76.6	Higher income predicted acceptance	19

AOR = adjusted odds ratio; NE = northeast; OR = odds ratio; SE = southeast; SES = socioeconomic status; SW = southwest.

There was a high level of willingness to vaccinate children, but a number of factors affected the desire for vaccination. Vaccine safety and side effects were the most common barriers reported in studies, as well as doubts regarding vaccine effectiveness. Other factors were socioeconomic constraints, level of education, cultural or religious factors, and family decision-making patterns. Other challenges are among the health care professionals, which include concerns about how vaccines were handled and the readiness of the health care system, including cold-chain integrity, that could affect vaccine delivery systems.^11^

Evidence of willingness to pay showed moderate financial readiness of caregivers, whereas affordability was a major factor in sustained uptake. In Enugu, 79.7% of respondents were willing to pay about USD $0.82 (₦639).^13^ The national survey also showed that although general acceptance of malaria vaccination was high, approximately 90% of the respondents were sensitive to cost (Figure 2), preferring the vaccine to be funded by the government, thus highlighting the importance of public financing mechanisms.^2^ The willingness to pay was also high in Lagos, where 71.2% of respondents indicated that they would be willing to pay, especially in the higher-income groups, with preferred payment ranges ranging from ₦1,001 to ₦5,000 per dose.^19^

The findings indicated that, overall, vaccination acceptance among the populations in Nigeria is high and generally favorable, but vaccine acceptance was influenced by vaccine safety concerns, vaccine effectiveness, socioeconomic concerns, and vaccine system confidence. Financial willingness was moderate yet heavily shaped by affordability, highlighting the crucial need for equitable financing strategies to ensure malaria vaccines were taken up and reached children.

### Health system readiness and facility preparedness.

One of the major challenges to the effective rollout of malaria vaccines in Nigeria is health system readiness. Using a WHO-validated readiness assessment framework, the preparedness for R21/Matrix-M introduction in three federal tertiary hospitals in Northern Nigeria was evaluated.^18^ The assessment examined multiple implementation domains, including health care worker training, vaccine supply and cold chain/logistics, advocacy and social mobilization, and monitoring and supervision. None of the facilities achieved the WHO-recommended preparedness threshold of 95%, indicating substantial implementation gaps before large-scale deployment.

The readiness score was low in all the facilities, with the Ahmadu Bello University Teaching Hospital (ABUTH), Zaria, having the highest score (51%), followed by the University of Maiduguri Teaching Hospital (UMTH) with 42% and the Jos University Teaching Hospital (JUTH) with 33%.^18^ Partial readiness was noted in areas like training and vaccine logistics (the UMTH was fully ready in cold chain and supply systems), but critical gaps were consistently identified in advocacy and social mobilization, monitoring, and supervision.^18^ The readiness assessment findings from tertiary hospitals in Northern Nigeria are presented in Table 4. The domains are key to successful vaccine uptake and post-introduction surveillance in immunization programs. All three hospitals, though, had a significant deficit in these areas, indicating that there is technical capacity to ensure vaccine storage and administration, but that gaps exist in community engagement and monitoring capabilities that may impede effective implementation and sustainability.

**Table 4:** R21 malaria vaccine readiness assessment at three Northern Nigerian tertiary hospitals

Readiness Domain	ABUTH Zaria	UMTH Maiduguri	JUTH Jos	Interpretation
Training	100% (full)	0% (off-track)	0% (off-track)	Only ABUTH achieved full readiness in training
Vaccine supply, cold chain, and logistics	60% (partial)	100% (full)	80% (partial)	Only UMTH was fully ready in logistics systems
Advocacy and social mobilization	0% (off-track)	0% (off-track)	0% (off-track)	All facilities critically underperforming
Monitoring and supervision	33% (off-track)	0% (off-track)	0% (off-track)	Weak post-implementation surveillance capacity
Overall readiness*	51%	42%	33% (off-track)	None met the WHO-recommended threshold of 95%

*Required threshold: ≥95% across all domains. Off-track = <50% readiness. ABUTH = Ahmadu Bello University Teaching Hospital; JUTH = Jos University Teaching Hospital; UMTH = University of Maiduguri Teaching Hospital.

### Comparative vaccine landscape and implementation implications.

Although both WHO-recommended malaria vaccines have the same public health goal, there are nuances to the operational and programmatic differences between R21/Matrix-M and RTS,S/AS01. R21/Matrix-M could potentially have benefits for broader deployment because of higher expected capacity for manufacturing and likely supply in the future, potentially enabling greater access in high-burden countries like Nigeria. In contrast, RTS,S/AS01 has been subjected to more intensive clinical testing, and long-term safety and efficacy data have been collected in several African countries.^20^ To date, however, RTS,S/AS01 has faced challenges in supply, while R21/Matrix-M is expected to be increasingly available because of the higher production capacity.^21^ Early implementation of R21/Matrix-M in Nigeria has offered valuable lessons for operational implementation, which will need to be analyzed for future scale-up of the program, though it is important to note that monitoring for long-term program performance is essential.^17^

## RECOMMENDATIONS

Based on the findings of this review, the implementation constraints identified in the existing literature found in Nigeria will have to be addressed for the successful nationwide rollout of malaria vaccines. The focus should first be on targeted public education campaigns to increase awareness among caregivers, especially in areas where the level of awareness is low, despite high levels of willingness to vaccinate. Communication efforts should focus on the safety and effectiveness of vaccines and the ongoing need for other malaria prevention tools.

Second, ongoing training should be offered for health care personnel, particularly nurses and pharmacists, to address stated gaps and concerns of vaccine safety, effectiveness, and handling. A boost in the confidence of health care workers will likely translate into the confidence of health care workers and uptake of vaccines.

Third, investments in health system strengthening should prioritize specific areas that have been repeatedly determined as lacking readiness in assessments such as advocacy, social mobilization, and monitoring and supervision. Greater improvements in these components will aid in program implementation and effective post-introduction monitoring.

Fourth, there is a need for sustainable financing mechanisms to make vaccines available to people in an equitable manner. The strong desire for government funding and moderate willingness to pay to vaccinate observed by caregivers will mean that continued government investments and support from international partners will be essential to sustain high vaccination coverage.

Finally, additional operational research is required to assess the effectiveness, safety, coverage, and implementation effects of the current R21/Matrix-M rollout in Nigeria. The evidence will guide future national expansion and help build evidence-based malaria vaccine policy.
